# Weak Anion Binding to Poly(*N*-isopropylacrylamide) Detected by Electrophoretic NMR

**DOI:** 10.1021/acs.jpcb.1c00642

**Published:** 2021-04-06

**Authors:** Yuan Fang, István Furó

**Affiliations:** Division of Applied Physical Chemistry, Department of Chemistry, KTH Royal Institute of Technology, SE-10044 Stockholm, Sweden

## Abstract

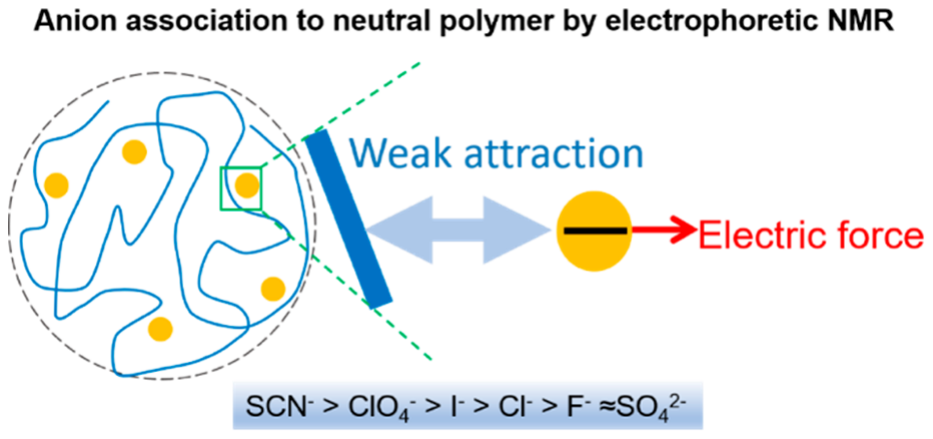

Ion specific effects are ubiquitous in solutions and govern a large number of colloidal phenomena. To date, a substantial and sustained effort has been directed at understanding the underlying molecular interactions. As a new approach, we address this issue by sensitive ^1^H NMR methods that measure the electrophoretic mobility and the self-diffusion coefficient of poly(*N*-isopropylacrylamide) (PNIPAM) chains in bulk aqueous solution in the presence of salts with the anion component varied from kosmotropes to chaotropes along the Hofmeister series. The accuracy of the applied electrophoretic NMR experiments is exceptionally high, on the order of 10^–10^ m^2^/(V s), corresponding to roughly 10^–4^ elementary charges per monomer effectively associated with the neutral polymer. We find that chaotropic anions associate to PNIPAM with an apparent Langmuir-type saturation behavior. The polymer chains remain extended upon ion association, and momentum transfer from anion to polymer is only partial which indicates weak attractive short-range forces between anion and polymer and, thereby and in contrast to some other ion–polymer systems, the lack of well-defined binding sites.

## Introduction

Salts dissociated into ions in water interact with colloids including biomolecules. As was found by Hofmeister and co-workers in the 1880s,^1^ those ions can be ordered by their ability to precipitate/stabilize proteins in solution. With regard to anions, this order is typically (depending on the phenomenon and the anions selected) as SO_4_^2–^ > H_2_PO_4_^–^ > F^–^ > Cl^–^ > Br^–^ > I^–^ > ClO_4_^–^ > SCN^–^ where anions on the left are prone to induce precipitation and usually termed as kosmotropes while the anions on the right called chaotropes increase protein stability in solution. The names reflect the original and by now surpassed hypothesis where the action of ions was connected to strengthening (kosmotropes) or weakening (chaotropes) the hydrogen bond network in bulk water.^2−6^ Today there are several, apparently overlapping ideas^7−10^ but no complete consensus concerning the Hofmeister effect, and therefore it is useful to apply to the problem new types of experimental techniques with new type of outcomes that may sharpen the test theories must endure.

Besides protein stability, a large number of other phenomena are also influenced by anions in similar apparent order. One example is the surface tension at the air–water interface. Irrespective of the phenomenon, the effect of ions hinges on their enrichment at or expulsion from interfaces, either macroscopic (air–water) or microscopic (biomolecule–water).^11−14^ In turn, the local interfacial concentration reflects the interactions of ions with the interfacial components relative to that with a bulk environment. As pointed out by diverse studies,^15−19^ the hydration of ions has a central role in defining those interactions. Yet, biomolecular surfaces are heterogeneous and often charged, and therefore there is an added complexity, particularly in comparison to the simple case of air (featuring in the surface tension and its Hofmeister behavior). As a noncharged model system with biomolecular relevance, poly(*N*-isopropylacrylamide) (PNIPAM) has often been studied. It has a simple composition where the side chains attached to the simple aliphatic backbone connected to a nonpolar isopropyl group via a polar acrylamide moiety. It is thermosensitive with a well-defined lower critical solution temperature (LCST) below which the extended polymer chain is soluble in water and above which the chain collapses into a less soluble globule. This feature, akin to protein denaturation, exhibits a strong Hofmeister effect.^17,20−23^ The Hofmeister effect with special relevance for this and other uncharged polymers was recently surveyed in a lurid and comprehensive manner.^9^ As discussed in detail there, ideas regarding interactions between ions and polymers are abound, but the impression remains that there is a need for more insight.

Here we detect the interaction of anions with PNIPAM using electrophoretic NMR (eNMR) in combination with diffusion NMR experiments at 20.0 °C, which is well below LCST. This combination of NMR techniques was recently used to quantify the effective charge of and thereby the strong cation binding to PEO in methanol.^24,25^ The first advantage of the method is being sensitive; its recently improved detection limit^26^ is significantly below a single elementary charge associated with the 79 kDa polymer (∼700 monomers). Second, the experimental principle is simple, and analysis can be performed without complex models. Third, in contrast to other electrophoretic detection methods, it requires no background electrolyte and/or pH buffer that may lend charge to small neutral entities.^27−29^ Finally, the technique works well at low salt (and polymer) concentrations where direct ion–solute interactions dominate and screening effects are less significant. Ion binding at PNIPAM-covered model surfaces has often been studied,^30−33^ but there is a lack of observations in dilute (here, at 10 mM monomer equivalent, therefore with no interactions between the extended chains) bulk solutions. The electrophoretic mobility μ of PNIPAM microgel particles was explored but only where the value of μ was much higher.^34,35^ This study contributes to further understanding of the direct interaction of anions with PNIPAM as a mechanism of specific ion effects and provides more insights regarding the molecular picture of ion binding.

## Materials and Methods

### Materials

**Figure 1:**
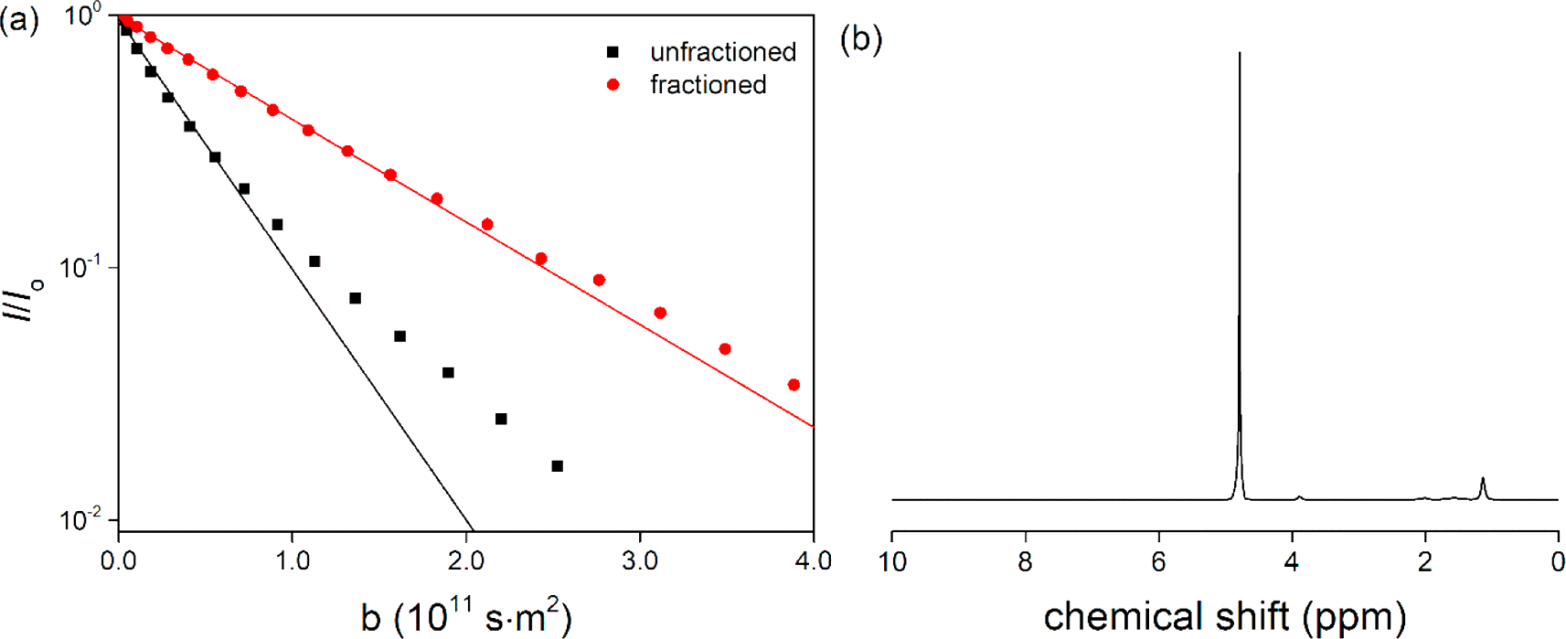
(a) ^1^H NMR diffusional decay of the methyl peak in the spectrum of PNIPAM either (■) as obtained or (●) fractioned. The solid line is a single-exponential fit to the initial echo attenuation that provides the average diffusion coefficicent *D*. (b) ^1^H NMR spectrum of the solution of the fractioned PNIPAM at 10 mM monomer-equivalent concentration in D_2_O at 20 °C. The diffusion and electrophoretic NMR experiments were performed on the PNIPAM methyl peak at around 1.1 ppm.

PNIPAM without any titratable groups was purchased from Polysciences, Inc. The polymer as purchased showed a considerable polydispersity so it was fractioned by phase separation using a mixture of dry acetone/*n*-hexane at room temperature according to the procedure described by Fujishige.^36^ All the fractions were dried under vacuum at 50 °C for 4 h before further characterization. The molecular weight distribution of different fractions was characterized by pulsed-field gradient NMR diffusion measurements performed at 10 mM monomer-equivalent concentration of PNIPAM in deuterated water (with the concentration established from the PNIPAM ^1^H NMR intensity; see the spectrum in Figure 1). This concentration is far below the overlap concentration, and polymer–polymer interactions were shown to have an insignificant influence on self-diffusion.^37^ The fraction that showed the narrowest molecular weight distribution (see below) was used throughout all experiments below. PNIPAM with charged end group was from Polymer Source, Inc. (*M*_n_ = 84000 g/mol with polydispersity index *M*_w_/*M*_n_ = 1.16).

All inorganic salts (from Sigma-Aldrich) were of analytical grade. All the other salts were dried under vacuum at 50 °C for at least 4 h before use. The PNIPAM solutions with 10 mM monomeric concentration were prepared the day before NMR experiments and were kept at 4 °C overnight to guarantee full dissolution. The water used was D_2_O (99.8% D).

### NMR Experiments

All ^1^H NMR experiments were performed on a Bruker Avance III 500 MHz spectrometer using a 5 mm DIFF30 probe which provided a maximum *z*-gradient strength of 1800 G/cm. The gradient current pulse was provided by a GREAT60 power supply unit. The experimental instrumentation including the eNMR 1000 electrophoretic power supply with current-control feature (P&L Scientific Instrument Service, www.plscientific.se) and the experimental procedures were described in great detail elsewhere.^26^ All the experiments were performed at 20.0 °C, which is well below the lower critical solution temperature (LCST) of PNIPAM with all salt species and concentrations used.

The diffusion NMR experiments were performed by using a double stimulated echo pulse sequence with the duration of the gradient pulses set to δ = 2 ms and the total diffusion time to Δ = 100 ms. The gradient strength *g* was incremented linearly from 2 to 230 G/cm in 16 steps, and the diffusion coefficient of PNIPAM was obtained by fitting the conventional Stejskal–Tanner expression
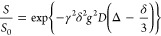
1to the variation of the NMR spectral integral upon increasing *g*. In eq 1, *S* and *S*_0_ are the integral intensities with and without gradient, and γ is the magnetogyric ratio. The gradient strength was calibrated by using the known value of trace ^1^H diffusion coefficient in heavy water.^26^ The obtained values are presented in Table 1.

**Table 1:** Self-Diffusion Coefficients of PNIPAM in Different Electrolyte Solutions in D_2_O

	*D* (10^–11^ m^2^/s)^a^					
*c* (M)	NaSCN	NaClO_4_	NaI	NaCl	NaF	Na_2_SO_4_
0.01	2.213	2.233	2.200	2.227		
0.05	2.200 ± 0.010	2.227	2.202	2.189	2.198	2.260
0.1	2.193	2.210	2.201	2.208		
0.2	2.198	2.267	2.245	2.191		
0.3	2.203	2.282	2.230	2.182		
0.4	2.199	2.275	2.215	2.221 ± 0.014		
no salt	2.215 ± 0.007					

a The stated errors are from experiments repeated in triplicate. The resulting conservative estimate of the error in diffusion experiments in this instrumental regime is on the order of ±0.7%.

In eNMR experiments, a double stimulated echo pulse sequence with bipolar electrophoretic voltages was used. The reference phase correction method previously described^26^ was used to obtain the phase ϕ of the NMR signal of the electromigrating species. In that method, the phase is measured relative to the phase of noncharged molecules, in our current case water. In eNMR experiments, the electric field *E* over the sample is stepped up incrementing the phase factor
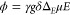
2from the slope of which μ can be extracted. An illustrative example of the raw phase data is presented in the Supporting Information (Figure S1). Recent extensive modifications of experimental setup (including cell design) and protocol^26^ permitted us to push accuracy to below 10^–10^ m^2^/(V s). The relative accuracy of the μ values obtained is below 2% and 3–5% for sample conductivity in the order of 1 and 10 mS/cm, respectively. Specifically, electric field pulses were implemented with controlling the current *I* that sets the electric field as *E* = π*r*^2^*I*/σ, where *r* is the tube radius and σ is the conductivity of the test solution. The eNMR measurements were performed by incrementing the current from −*I*_0_ to *I*_0_ in 13 steps (with δ = 2 ms and Δ = 100 ms) while keeping gradient strength constant at *g* = 23 G/cm. The maximum current *I*_0_ applied across the sample cell was 20, 60, 110, 110, 60, and 60 mA for 10, 50, 100, 200, 300, and 400 mM salt solutions, respectively. A slight temperature increase due to Joule heating was noticed and corrected for.^26^

The precision of measurements was evaluated with three independent repetitions for the samples containing NaSCN and NaCl. In combination with the error of diffusion experiments, this sets our detection limit of effective charge (see below) to 1.5 × 10^–4^ elementary charge per PNIPAM monomer in the ≤100 mM regime. At higher concentrations (≥300 mM) with higher sample conductivity (see Table S1), thermal convection due to sample heating limited the current value to maximum 60 mA (see above). The lower available current and the higher conductivity yielded a lower electric field *E* and thereby lower μ*E* drift velocities.^26^ Hence, the experimental phase factors (see Figure S1) were reduced, and the fitting errors for the μ values have increased.

**Table 2:** Electrophoretic Mobilities of PNIPAM in Different Electrolyte Solutions in D_2_O

	μ (10^–9^ m^2^/(V s))			
*c* (M)	NaSCN	NaClO_4_	NaI	NaCl
0.01	0.75	0.52	0.78	0.29
0.05^a^	1.79 ± 0.09	1.17	1.30	0.41 ± 0.08
0.1	2.87	2.15	2.32	0.72
0.2^a^	3.65 ± 0.30	2.51	2.84	1.14
0.3^b^	3.88 ± 0.29	3.26 ± 0.23	3.26 ± 0.26	
0.4^b^	5.51 ± 0.64	3.75 ± 0.28	4.04 ± 0.45	

a Errors from experiments repeated in triplicate.

b Fitting errors caused by increased scatter of the experimental phase factors.

### Conductivity Measurements

Conductivity measurements (see Table S1) were performed with a CDM210 conductivity meter (Radiometer Analytical, Copenhagen) and a two-pole conductivity cell CDC749. The temperature of the sample cell was controlled at 20.0 °C by a cryostat (H F4-Q with Synth 60 bath liquid) that provided a stable temperature from −50 to 45 °C with ±0.03 °C accuracy. The cell constant was calibrated with either 10 or 100 mM aqueous solution of KCl at 20.0 °C.

## Results and Discussion

### Monodisperse PNIPAM without Any Titratable End Group

The PNIPAM used here was carefully fractionated to provide a narrow size distribution. The narrowness of the molecular size distribution was confirmed by diffusion NMR experiments (see Figure 1). As is shown by eq 1, polymer chains of the same size and thereby the same diffusion coefficient *D* exhibit a single-exponential signal decay ∝ exp (−*bD*), where *b* = γ^2^δ^2^*g*^2^(Δ – δ/3) while polydisperse solutions provide multiexponential decays. As demonstrated by Figure 1, fractioning very much reduced the polydispersity of PNIPAM. The diffusion coefficient measured for fractioned PNIPAM was *D* = 2.215 × 10^–11^ m^2^/s, from which the average molecular weight was estimated to *M*_w_ = 7.9 × 10^4^ g/mol which corresponds to ∼700 monomers per average polymer.^38^

**Figure 2:**
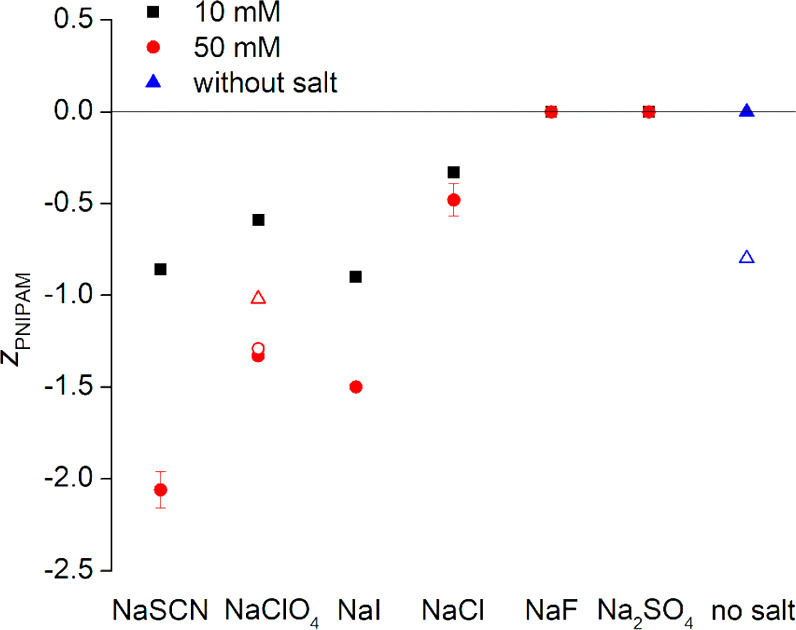
Effective charge of PNIPAM molecules at 10 mM monomeric concentration in water without salt (▲) and with 10 mM (■) or 50 mM added salt (●), all at 20.0 °C. The obtained effective charge of PNIPAM with one carboxylate end group per polymer (blue △) and the effective charge values with 50 mM LiClO_4_ (○) and N(CH_3_)_4_ClO_4_ (red △) are also indicated. The error bars indicate precision as derived from triplicate experiments. The effective charge values with no salt, with NaF and with Na2SO4 were all within the ±0.04 range.

Depending on the selected polymerization method, PNIPAM may be created with a carboxylate end group, a feature that is sometimes ignored in the literature. Because in this study we wished to investigate weak ionic effects, it was of utmost importance to clarify the state of PNIPAM in this respect. For the experiments below, we thereby specifically chose a PNIPAM without any titratable groups even at the end of chains. Moreover, to perform a positive test of neutrality, we also purchased PNIPAM with a carboxylate end group (from Polymer Source, Inc.). In a salt-free solution at neutral pH, PNIPAM with no titratable groups was expected to be neutral and provide zero electrophoretic mobilty and thereby zero effective charge (see eq 3). Indeed, this was found as shown by Figure 2. On the other hand, PNIPAM with a carboxylate end group was expected to provide an effective charge close to −1 (reduced somewhat in magnitude by charge screening); indeed, the experimental effective charge is −0.8 ± 0.1 (see Figure 2). Hence, our results below reflect truly the weak anion interactions by a neutral polymer.

### Chaotropic Anions Bind While Kosmotropic Anions Do Not

The diffusion coefficients and the electrophoretic mobilities were measured and used, through the Nernst–Einstein relation, to obtain the effective charge (in units of elementary charge) that was attained by the PNIPAM molecule upon association to it by ions
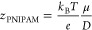
3where *k*_B_ represents the Boltzmann factor, *T* the absolute temperature, and *e* the elementary charge. The values obtained are shown in Figure 2.

Clearly, neat PNIPAM is neutral and remains so with NaF and Na_2_SO_4_ added. However, in the presence of NaSCN, NaClO_4_, NaI, and NaCl, PNIPAM attains a negative charge. This is direct evidence that chaotropic anions preferably associate to PNIPAM while kosmotropic anions do not. PNIPAM being neutral with NaF and Na_2_SO_4_ also indicates that the association of Na^+^ to PNIPAM is negligible as is expected for a “borderline” ion.^9^ Experiments with other cations (Figure 2) show that the effective charge is less affected by changing cation species, yet the large, polarizable, and weakly hydrated N(CH_3_)_4_^+^ ion clearly exhibits some binding. It was implied previously^39^ that ion specific effects only appear at high concentration, at least 100 mM or above. This is clearly not the case.

**Figure 3:**
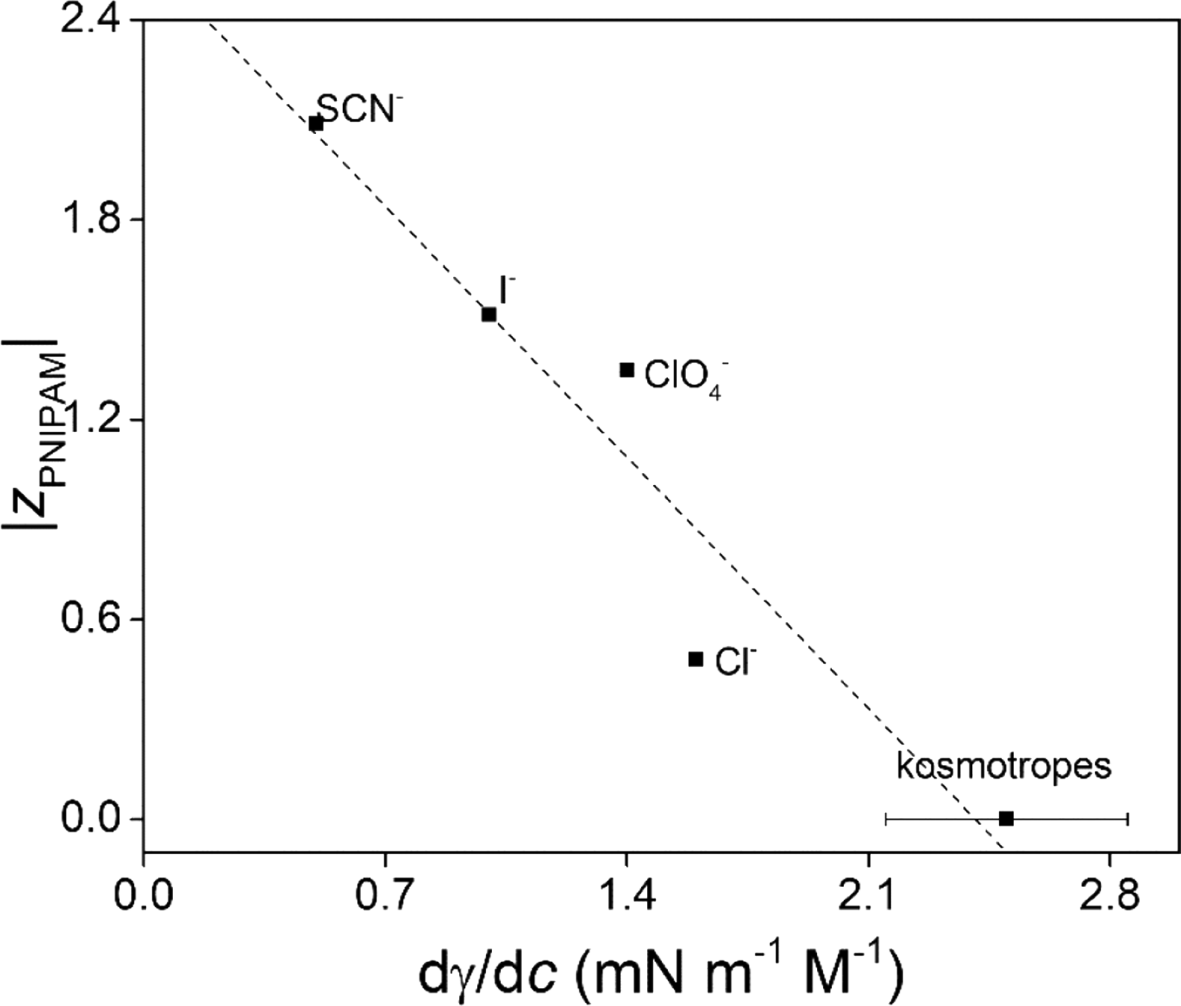
Obtained effective charge of PNIPAM plotted against the surface tension increment (that is, as compared to the average over kosmotropes F^–^, H_2_PO_4_^–^, S_2_O_3_^2–^, SO_4_^2–^, and CO_3_^2–^) for salts at 50 mM. The dotted line is a linear fit.

Although the anion association is extremely weak, the differences between salts are significant. Anion accumulation in the proximity of PNIPAM was reported both in bulk and at the interface but usually indirectly. Roughly the same anion order was reported previously by other techniques with some disagreement over the relative positions of thiocyanate and perchlorate anions,^17,21,40−42^ although most previous observations were not quantitative. Chloride ions were often described as nonassociated, and this point is disproved by results here obtained by a method of superior sensitivity. In Figure 3, we present the correlation between the obtained effective charge with the surface tension data^43^ at the air–water interface. Some studies have shown that macromolecule structure as well as molecular weight and concentration influence the magnitude of Hofmeister effects.^18,44,45^ In contrast, the correlation presented in Figure 3 seems to indicate that molecular details may matter little regarding the interaction trends of chaotropic anions with neutral interfaces at which polarity drops.^46^

### Quantitative Ion Binding

**Figure 4:**
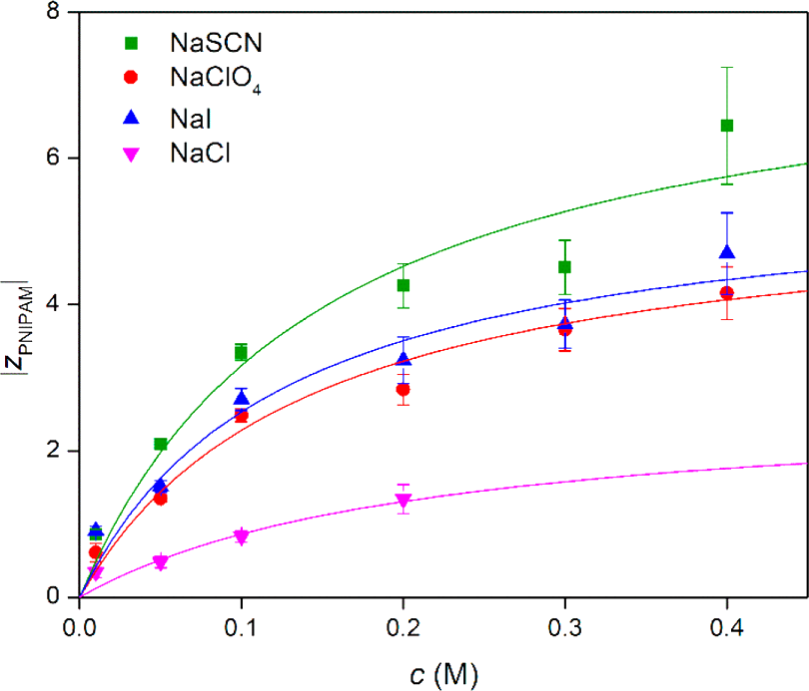
Effective charge of PNIPAM upon increasing salt concentration. The solid lines are least-squares fits of the Langmuir isotherm model in eq 4 to the data.

The association of a few single ions to a large polymer like the PNIPAM is not well represented by assigning a small surface charge density spread over a rather large particle representing the polymer (a notion customary in colloidal electrophoresis^47^). Instead, the electric field remains undistorted as it interacts with the ions residing in the well-hydrated volume of the extended coil. Hence, the effective charge should represent well (contribution from counterion binding should be small at our lowest concentrations, where the Debye length of 3 nm is larger than Bjerrum length, 0.7 nm) the actual charge of the ion-decorated chain. Yet, the tacit assumption here is that the ion–polymer interaction is strong enough to provide full momentum transfer^48−50^ from the ion acted upon by the electric field to the polymer. This was clearly the case for cations binding to poly(ethylene oxide) (PEO) in methanol.^24^ Because the anion association (as indicated by Figure 2) is weak, this assumption may not be justified, and if so, the experimental effective charge is reduced. This effect is surprisingly not well investigated. Hence, ion association may not be quantified directly from the magnitude of the obtained effective charge, and therefore the dependence of the effective charge upon concentration is investigated (Figure 4).

The data conform to binding isotherms whose shape is in rough agreement with those from other observations^17,31^ and can be fitted well with a Langmuir isotherm
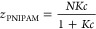
4where *N* is the maximum effective charge per molecule, *K* is the binding constant, and *c* is the molar concentration of salt. The outcome listed in Table 3 includes the indicative value of the free energy of binding deduced from the (most probable) value of *K*. The *NK* product that is plausibly suggestive of the extent of the binding is largest for the SCN^–^ anion that is often named in the literature as the one exhibiting the strongest binding.

**Table 3:** Binding Constant *K* and Maximum Effective Charge *N* Were Obtained from the Data in Figure 4

	*K* (M^–1^)	*N*	Δ*G*^a^ (kJ/mol)
SCN^–^	6.7 ± 3.0	7.9 ± 1.4	–4.6
ClO_4_^–^	7.3 ± 2.1	5.4 ± 0.6	–4.8
I^–^	8.0 ± 2.9	5.7 ± 0.6	–5.1
Cl^–^	4.7 ± 4.3	2.7 ± 1.5	–3.8

a Δ*G = −k*_B_*T* ln(*K*).

On one hand, the results in Figure 4 and Table 3 confirm the binding behavior for chaotropes to PNIPAM concluded by Cremer et al.^17,21,31^ from phase transition shift in bulk solutions and from surface potential data at surfaces. The binding constants and the derived free energies of binding are close to values previously deduced (and in a similar order as that for other molecular surfaces).^20,40,51−53^ Binding constants can also be derived by using a bulk-surface partition model^12^ (see the Supporting Information). As another alternative, binding can be interpreted via the preferential interaction parameter within the Kirkwood–Buff theory^54^ (see the Supporting Information).

**Figure 5:**
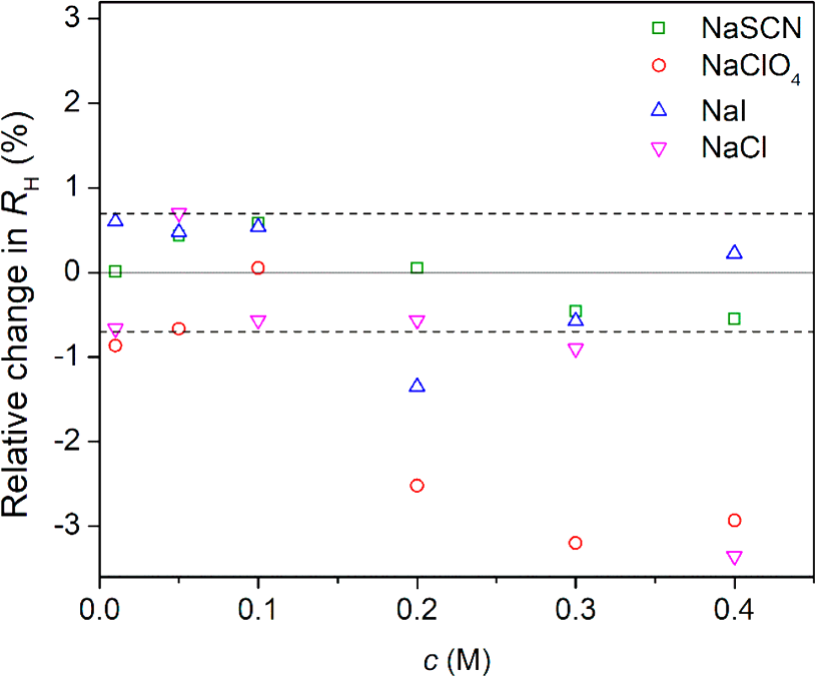
Change of hydrodynamic radius *R*_H_ of PNIPAM in the presence of different salts relative to that in pure water (with *R*_H_ = 7.75 nm). The viscosity of salt solutions was obtained via the Jones–Dole viscosity coefficients and included in the Stokes–Einstein equation (see the Supporting Information). The dashed lines indicate the range corresponding to the experimental error (±σ); hence, points outside that range may indicate significant changes in *R*_H_.

In discussing the implications of the data for the nature of binding, we also rely on our diffusion data transformed into hydrodynamic radii in Figure 5. As the outset, we recapitulate that single-charged cations K^+^, Rb^+^, and Cs^+^ that strongly (with a binding constant of approximately *K* = 500) bind to PEO in methanol make the polymer chain to shrink (at 2 mM salt, the *R*_H_ of the PEO chain decreases by about 10–15%)^24^ which was attributed to the chain being locally wrapped about the cation, thereby providing a binding site.^25^ Clearly, the accumulation of most anions around the PNIPAM chain is not associated with chain reorganization as is shown by the constancy of *R*_H_ over salts at different concentrations. Altogether, the notion of “binding site” is difficult to reconcile with the values of *N* in Table 3 which would imply saturation at the level of one extra anion per hundred monomers (recall that the investigated PNIPAM is ∼700 monomers). Yet (i) *N* is much lower for Cl^–^ than that for the other anions and (ii) with one extra anion per hundred monomers there is indeed no mechanism that would yield a saturation behavior. Hence, we must invoke that, in contrast to cations associated in methanol with PEO,^24,25^ the actual attractive interaction between anions and PNIPAM is so weak that the momentum transfer from ion to polymer is only partial.^48−50^ If so, the actual number of ions can be high enough to render ion–ion repulsion as a mechanism limiting the number of anions associated with a PNIPAM chain (for example, with 20 ions per chain, the anion–anion distance approaches 1 nm). Thus, *N* being lower for Cl^–^ becomes the consequence of the attractive short-range forces between Cl^–^ and PNIPAM being weaker than that for the other chaotropic anions (leading to smaller momentum transfer). This goes well in line with Cl^–^ exhibiting a lower dispersion potential.^11^ Nevertheless, the notion of weak attractive short-range forces between anions and PNIPAM suggests that anion accumulation around PNIPAM is of a rather diffuse character instead of being accomplished at “binding sites”.

As a minor observation, NaCl at 400 mM slightly shrinks the PNIPAM chain that we suggest is the sign of the chain becoming less extended, a pretransitional behavior in combination with NaCl decreasing the phase transition temperature. The perchlorate ion behavior is slightly different from that of the other anions as it seems to induce a slight shrinkage of the polymer chain. The large size of perchlorate has been previously implicated^31,55,56^ in causing some anomalies; we suggest that wrapping the chain around a large ion is entropically less costly than doing that around a smaller ion. Finally, recently^57^ and similarly to that in the methanol–PEO system,^24,25^ ion pairs were indicated to play a role in anion binding to PNIPAM. Being neutral, the presence of tightly bound pairs would be missed here by our method.

## Conclusions

In summary, we have shown that anion binding to a neutral polymer PNIPAM in bulk aqueous solution can be characterized by a combination of electrophoretic and diffusion NMR methods. We confirm previous observations by very different and, in comparison to ours here, less direct methods that recorded a Langmuir-type association behavior where the binding constant for chaotropic anions roughly follows the Hofmeister order. On the other hand, the low apparent saturation level indicates that momentum transfer from ion to polymer that is only partial, which in turn indicates weak short-range attractive forces between ion and polymer. This suggests that the surplus of anions around the PNIPAM molecule is manifested more as a diffuse cloud rather than occupying specific binding sites. The lower apparent saturation level observed for Cl^–^ becomes then the consequence of the lower dispersion potential of Cl^–^ as compared to that of I^–^, ClO_4_^–^, and SCN^–^.

## Supporting Information Available

Raw eNMR phase data; conductivities; hydrodynamic radii of PNIPAM *R*_H_; data evaluation within the bulk-surface partition model and the Kirkwood–Buff model (PDF)
