# Supplementary material for: Weak Anion Binding to Poly(N-isopropylacrylamide) Detected by Electrophoretic NMR
Source: J Phys Chem B. 2021 Apr 6;125(14):3710–6. doi: 10.1021/acs.jpcb.1c00642 (PMC8154593; doi:10.1021/acs.jpcb.1c00642)
Supplement: Supplementary file 1 — jp1c00642_si_001.pdf [file jp1c00642_si_001.pdf]

Supporting Information

for

Weak Anion Binding to Poly(N-Isopropylacrylamide) Detected by Electrophoretic NMR

Yuan Fang and István Furó*

[a]Division of Applied Physical Chemistry, Department of Chemistry, KTH Royal Institute of Technology, SE-10044 Stockholm, Sweden

Table of Contents

Typical raw data of eNMR..2

Table S1. The conductivities of PNIPAM in different electrolyte solutions in D_2O. 2

The hydrodynamic radii of PNIPAM R_H at different salt concentrations 2

Table S2. Jones-Dole viscosity coefficients of aqueous electrolyte at 20.0 °C. 3

Table S3. The hydrodynamic radius of PNIPAM at different concentrations of salts........................3

Data evaluation within the bulk-surface partition model and the Kirkwood-Buff model...................3

Table S4. The partition coefficients of anions in PNIPAM solutions of different electrolyte concentrations. The right column is relevant partition coefficients from the literature. 4

Table S5. The free energy of anion binding to PNIPAM obtained from both Langmuir isotherm model and solute partition model...5

Typical raw data of eNMR

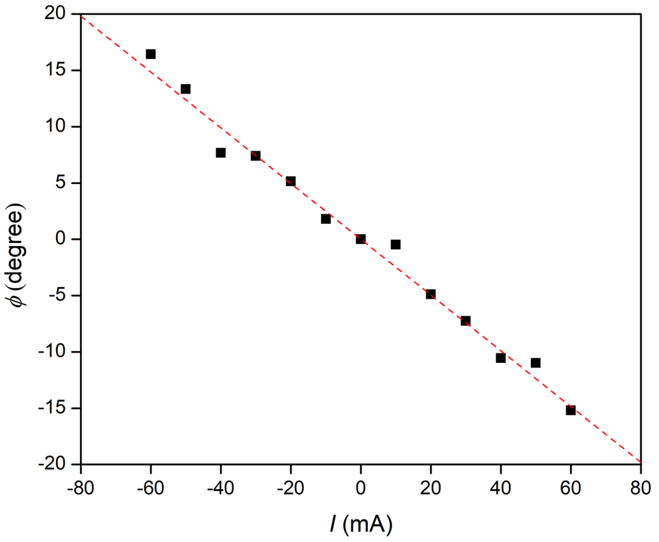

Figure S1. The phase difference ϕ of PNIPAM ^{1}H NMR peaks relative to the HOD peak at different currents I. The sample is PNIPAM at 10 mM monomeric concentration in D_2O with 50 mM NaSCN added.

Table S1. The conductivities of PNIPAM in different electrolyte solutions in D_2O.

c (M)	σ (mS/cm)					
	NaSCN	$NaClO_4$	NaI	NaCl	NaF	Na_2SO_4
0.01	0.736	0.725	0.872			
0.05	3.301	3.437	3.508	3.557	2.198	6.720
0.1	7.480	7.510	8.100	7.990		
0.2	13.920	13.840	15.260	15.070		
0.3	19.570	19.520	21.560	20.950		
0.4	25.330	24.480	27.590	25.690		

The hydrodynamic radii of PNIPAM R_H at different salt concentrations

PNIPAM as a random coil with hydrodynamic radii R_H with the diffusion coefficient related to viscosity η by Stokes-Einstein equation

$$D = \frac{k_B T}{6\pi\eta R_H}. \tag{S1}$$

Since there are specific ion effects on viscosity, in order to compare PNIPAM chain conformation in different electrolytes and at different concentrations these effects need to be corrected for. The viscosity of an electrolyte at concentration *c* can be approximated by the Jones-Dole expression

$$\frac{\eta}{\eta_0} = 1 + A\sqrt{c} + Bc \qquad \text{(S2)}$$

where η_0 is the viscosity of pure solvent, see Table S2. The *A* coefficients in H_2O are known from literature at 25 °C and their values at 20 °C were estimated as $A \propto \frac{1}{\eta_o \sqrt{T}}$ [1]. The viscosity of water is from reference.[2] The *B* coefficients in H_2O at 20°C were directly available from literature. Values used are listed in the Table S2 and the references indicated. For calculating the hydrodynamic radii R_H, the diffusion coefficients of PNIPAM measured in D_2O were finally transformed to equivalent diffusion coefficients in H_2O (for which the Jones-Dole coefficients are known) by the relation $D(D_2O)\eta_0(D_2O) = D(H_2O)\eta_0(H_2O)$.

Table S2. Jones-Dole viscosity coefficients of aqueous electrolyte at 20.0 °C.

	A*($dm^{3/2} \cdot mol^{-1/2}$)	B ($dm^3 \cdot mol^{-1}$)
NaSCN	0.0003[3]	0.032[4]
$NaClO_4$	0.0061[5]	-0.002[1]
NaI	0.0097[6]	-0.021[1]
NaCl	0.0056[1]	0.071[1]

* from reference the viscosity A coefficient is for 25.0 °C.

Table S3. The hydrodynamic radius of PNIPAM at different concentrations of salts.

c (M)	R_H(nm)			
	NaSCN	$NaClO_4$	NaI	NaCl
0		7.75		
0.01	7.75	7.68	7.80	7.70
0.05	7.78	7.70	7.79	7.80
0.1	7.80	7.75	7.79	7.71
0.2	7.75	7.55	7.64	7.71
0.3	7.71	7.50	7.71	7.68
0.4	7.71	7.52	7.77	7.49

Data evaluation within the bulk-surface partition model and the Kirkwood-Buff model

The free energy change as result of association/binding can also be evaluated in terms of the solute partition model,[7-10] frequently used to rationalize ion-specific surface tension behavior with direct

Hofmeister order but also binding studies in in peptides and biomembranes. There, the free energy change is related with the relative concentration of anions in direct vicinity of PNIPAM c_{in} and in the bulk c by

$$\frac{c_{in}}{c} = e^{-\frac{\Delta G}{k_B T}} \qquad \text{(S3)}$$

This ratio is often referred to as the partition coefficient *K*p defining the concentration of ions at the solution-polymer interface relative to their concentration in the bulk solution.[8, 9] It is important to note that individual *K*p values reported depend on the assumed interface thickness, usually considered to be two layers of water molecules (6 Å). On the other hand, computational studies typically show that the concentration profile of anions and cations in vicinity of PNIPAM reach the bulk concentration beyond 1-3 nm.[11-13] Hence, it is worth to point out that numerical values of *K*p are highly dependent on the underlying assumptions.

Here we define the hydrodynamic volume as the boundary of PNIPAM surface and the bulk solution. Thus the anion concentration inside the hydrodynamic volume can be calculated as $c_{in} = \frac{N_{in}}{V} = \frac{cV + z_{PNIPAM}(c)/N_A}{V}$, where $V = \frac{4}{3}\pi R_H{}^3$ is the hydrodynamic volume, *c* is the bulk salt concentration and N_A is the Avogadro constant. Hydrodynamic radii R_H can be obtained from diffusion coefficients by Stokes-Einstein equation. A similar model was used to evaluate solute binding to PNIPAM by molecular dynamic simulations recently.[10] The partition coefficients *K*p as well as the free energy of partition estimated according to eq (S3) were summarized in Table S4 and S5. As discussed above, we cannot compare directly our *K*p values to those presented in the literature.[12, 13] However, the obtained free energy of solute to PNIPAM by solute partition model is in the same magnitude as that by Langmuir isotherm model.

Table S4. The partition coefficients of anions in PNIPAM solutions of different electrolyte concentrations. The right column is relevant partition coefficients from the literature.

	*K*p[a]						*K*p[b]
	0.01 M	0.05 M	0.1 M	0.2 M	0.3 M	0.4 M	
NaSCN	27.3	13.1	10.7	7.3	5.6	5.9	1.64
$NaClO_4$	18.8	9.1	8.3	5.5	4.9	4.4	1.83
NaI	27.2	9.8	8.7	5.9	4.7	4.3	1.18
NaCl	11.4	3.9	3.5	2.9	-	-	0.70

a. data from this work.

b. data from the reference 8.

Table S5. The free energy of anion binding to PNIPAM obtained from both Langmuir isotherm model and solute partition model.

	$\Delta G_{Langmuir}$ (kJ/mol)	ΔG_{SPM} (kJ/mol)[a]
NaSCN	-4.6	-6.3
$NaClO_4$	-4.8	-5.4
NaI	-5.1	-5.6
NaCl	-3.8	-3.2
NaF	0.0	0.0

a. from data recorded at 0.05 M.

The weak interaction of electrolytes to macromolecules can be described by the preferential interaction parameter in classical solution theory.[14] In a three-component system. with the solvent (water) being (1). the macromolecule (2) and the cosolvent (salt) (3). the preferential interaction parameter is defined as

$$\Gamma_{23} = \left(\frac{\partial m_3}{\partial m_2} \right)_{T,\mu_1,\mu_3} \qquad \text{(S4)}$$

where m_2 and m_3 are molar concentrations of macromolecule and salt. respectively. This equation is strictly valid in an open system at infinitely low macromolecular concentration. The Kirkwood-Buff (KB) theory is often used in evaluating the preferential interaction parameter since it provides a path to relate computational results to experimental thermodynamic data. The KB integrals between species *i* and *j* are defined as $G_{ij} = G_{ji} = 4\pi \int_0^\infty \left[g_{ij}^{\mu VT}(r) - 1 \right] r^2 dr$. where $g_{ij}(r)$ is the radial distribution function between species *i* and *j*. With the number density (molar concentration) of species *i* $\rho_i = \frac{N_i}{V}$ we obtain $N_{ij} = \rho_j G_{ij}$ as the excess number of *j* molecules around a single *i* molecule relative to its reference solution. From eq (S4) and the KB integrals one can obtain $\Gamma_{23} = \frac{\rho_3 (G_{23} - G_{21})}{1 + \rho_2 (G_{22} - G_{21})}$.[15] At infinite dilution. this simplifies to $\Gamma_{23} = \rho_3 (G_{23} - G_{21}) = N_{23} - \frac{\rho_3}{\rho_1} N_{21}$.[14-17] A negative Γ_{23} means that the co-solutes are depleted in the interfacial region and a positive Γ_{23} means accumulation of co-solutes. Since our salt and polymer concentrations are low. we assume that this leads to $\Gamma_{23} = N_{23} - \frac{m_3}{m_1} N_{21}$ where $\frac{\rho_3}{\rho_1} = \frac{m_3}{m_1}$ is the bulk ratio of salts to solvent. This is the actual excess number of co-solute in vicinity of polymer and- if the effective charge is a measure of that- it provides a way of interpreting z_{PNIPAM}.

Supporting References

(1) Jenkins, H.D.B.; Marcus, Y. Viscosity B-coefficients of ions in solution. *Chem. Rev.* **1995,** *95,* 2695-2724.

(2) Hardy, R.C.; Cottington, R.L. Viscosity of deuterium oxide and water in the range 5 to 125 °C. *J. Res. Natl. Bur. Stand., Sect. B* **1949,** *42,* 573.

(3) Oncsik, T.; Trefalt, G.; Borkovec, M.; Szilagyi, I. Specific ion effects on particle aggregation induced by monovalent salts within the Hofmeister series. *Langmuir* **2015,** *31,* 3799-3807.

(4) Tsierkezos, N.G.; Molinou, I.E. Viscosity coefficients of NaSCN, KSCN, NH_4SCN and $MnSO_4$, $CoSO_4$, $NiSO_4$, $CdSO_4$ in aqueous binary mixtures of N,N-dimethylformamide at 20 °C. *Z. Phys. Chem* **2004,** *218,* 211-218.

(5) Nightingale, E.R. Viscosity of aqueous sodium perchlorate solutions. *J. Phys. Chem.* **1959,** *63,* 742-743.

(6) Abdulagatov, I.M.; Zeinalova, A.B.; Azizov, N.D. Viscosity of aqueous electrolyte solutions at high temperatures and high pressures. Viscosity B-coefficient. sodium iodide. *J. Chem. Eng. Data* **2006,** *51,* 1645-1659.

(7) Wimley, W.C.; White, S.H. Membrane partitioning: distinguishing bilayer effects from the hydrophobic effect. *Biochemistry* **1993,** *32,* 6307-6312.

(8) Pegram, L.M.; Record, M.T. Hofmeister salt effects on surface tension arise from partitioning of anions and cations between bulk water and the air-water interface. *J. Phys. Chem. B* **2007,** *111,* 5411-5417.

(9) Pegram, L.M.; Record Jr, M.T. Thermodynamic origin of Hofmeister ion effects. *J. Phys. Chem. B* **2008,** *112,* 9428-9436.

(10) Kanduč, M.; Chudoba, R.; Palczynski, K.; Kim, W.K.; Roa, R.; Dzubiella, J. Selective solute adsorption and partitioning around single PNIPAM chains. *Phys. Chem. Chem. Phys.* **2017,** *19,* 5906-5916.

(11) Okur, H.I.; Hladílková, J.; Rembert, K.B.; Cho, Y.; Heyda, J.; Dzubiella, J.; Cremer, P.S.; Jungwirth, P. Beyond the Hofmeister series: ion-specific effects on proteins and their biological functions. *J. Phys. Chem. B* **2017,** *121,* 1997-2014.

(12) Micciulla, S.; Michalowsky, J.; Schroer, M.A.; Holm, C.; von Klitzing, R.; Smiatek, J. Concentration dependent effects of urea binding to poly(N-isopropylacrylamide) brushes: a combined experimental and numerical study. *Phys. Chem. Chem. Phys.* **2016,** *18,* 5324-5335.

(13) Rodríguez-Ropero, F.; van der Vegt, N.F.A. Direct osmolyte–macromolecule interactions confer entropic stability to folded states. *J. Phys. Chem. B* **2014,** *118,* 7327-7334.

(14) Smith, P.E. Chemical potential derivatives and preferential interaction parameters in biological systems from Kirkwood-Buff theory. *Biophys. J.* **2006,** *91,* 849-856.

(15) Smith, P.E. Cosolvent interactions with biomolecules: relating computer simulation data to experimental thermodynamic data. *J. Phys. Chem. B* **2004,** *108,* 18716-18724.

(16) Smith, P.E. Equilibrium dialysis data and the relationships between preferential interaction parameters for biological systems in terms of Kirkwood−Buff integrals. *J. Phys. Chem. B* **2006,** *110,* 2862-2868.

(17) Heyda, J.; Dzubiella, J. Thermodynamic description of Hofmeister effects on the LCST of thermosensitive polymers. *J. Phys. Chem. B* **2014,** *118,* 10979-10988.
